# Comparison of lactate/albumin ratio to lactate and lactate clearance for predicting outcomes in patients with septic shock admitted to intensive care unit: an observational study

**DOI:** 10.1038/s41598-022-14764-z

**Published:** 2022-07-29

**Authors:** Kamran Shadvar, Nader Nader-Djalal, Noushin Vahed, Sarvin Sanaie, Afshin Iranpour, Ata Mahmoodpoor, Amir Vahedian-Azimi, Abbas Samim, Farshid Rahimi-Bashar

**Affiliations:** 1 Department of Anesthesiology and Intensive Care Medicine, Faculty of Medicine, Tabriz University of Medical Sciences, Tabriz, Iran; 2 Department of Anesthesiology, Buffalo University, Buffalo, NY USA; 3 Faculty of Medicine, Tabriz University of Medical Sciences, Tabriz, Iran; 4 Neurosciences Research Center, Aging Research Institute, Tabriz University of Medical Sciences, Tabriz, Iran; 5 Al-Zahra Private Hospital, Dubai, UAE; 6 Evidence Based Research Center, General ICU, Shohada Hospital, Tabriz University of Medical Sciences, Gol Gasht Street, Tabriz, Iran; 7 Trauma Research Center, Nursing Faculty, Baqiyatallah University of Medical Sciences, Tehran, Iran; 8 Chemical Injuries Research Center, Systems Biology and Poisonings Institute, Baqiyatallah University of Medical Sciences, Tehran, Iran; 9 Anesthesia and Critical Care Department, Hamadan University of Medical Sciences, AyatolahMotahari BLVD, Resalat Square, Hamadan, 6514845411 Iran

**Keywords:** Biomarkers, Medical research

## Abstract

The aim of this study was to evaluate the prognostic value of the Lactate to Albumin (L/A) ratio compared to that of lactate and lactate clearance in predicting outcomes in patients with septic shock. This was a multi-center observational study of adult patients with septic shock, who admitted to intensive care units (ICUs) at Shohada and Imam Reza Hospitals, Tabriz, Iran, between Sept 2018 and Jan 2021. The area under the curve (AUC) of receiver operating characteristic (ROC) curve and multivariate logistic regression analyses were used to explore associations of the L/A ratio, lactate and lactate clearance on the primary (mortality) and secondary outcomes [ICU length of stay (LOS), duration of mechanical ventilation (MV), need of renal replacement therapy (RRT) and duration of using vasopressors] at baseline, 6 h and 24 h of septic shock recognition. Best performing predictive value for mortality were related to lactate clearance at 24 h, L/A ratio at 6 h and lactate levels at 24 h with (AUC 0.963, 95% CI 0.918–0.987, *P* < 0.001), (AUC 0.917, 95% CI 0.861–0.956, *P* < 0.001), and (AUC 0.904, 95% CI 0.845–0.946, *P* < 0.001), respectively. Generally, the lactate clearance at 24 h had better prognostic performance for mortality and duration of using vasopressor. However, the L/A ratio had better prognostic performance than serum lactate and lactate clearance for RRT, ICU LOS and MV duration.

## Introduction

Sepsis and septic shocks remain a major burden on the health care system which is responsible for approximately 30 to 40% of in-hospital mortality and an overall mortality rate of almost 25%^1,2^. The most important point regarding sepsis management is early recognition and appropriate management^3^. Management delays have been associated with increased mortality and morbidity^4,5^. Rapid detection of high-risk patients remains a challenge, and several attempts have been made to identify readily available and cost-effective biomarkers to predict septic patients and stratify risk^6,7^.

Lactate has been studied over time in patients with septic shock, and serum lactate is an important prognostic factor that reflects decreased oxygen delivery and tissue hypoperfusion^8–10^. The finding of hyperlactatemia in patients with normal tissue perfusion and oxygen delivery additionally suggests that an overstimulation of the Na+–K+–ATPase leads to an increased lactate production in septic patients similar to hypokalemia. Current recommendation is to measure serum lactate levels in all patients with suspected sepsis within 1 h of presentation and repeat measurements within 2–4 h if the initial lactate level exceeds 2 mmol/L^11^. However, increased lactate levels can be due to number of different etiologies like decreased lactate elimination due to hepatic or renal dysfunction as well as accelerated glycolysis, taking certain medications such as metformin, albuterol, some comorbidities including diabetic ketoacidosis, malignancies and also as a consequence of epinephrine infusion^12–18^. Hence, relying solely on the lactate levels may be inadequate. In addition, evidence shows that the lactate clearance at the first hours was associated with an improvement of outcome in severe sepsis and septic shock patients and was proposed to guide treatment^9,19–23^. Reduced lactate clearance may reflect globally impaired renal and hepatic metabolic function, both of which normally contribute to systemic lactate disposal. Thus, lactate clearance biologically reflects homeostasis of the host and provides more meaningful data about the overall adequacy of the resuscitative processes^19,24^.

Other emerging sepsis biomarker is the lactate to albumin (L/A) ratio. Albumin as a negative acute-phase protein can also serve as a biomarker for prognosis in septic patients^25^. Given that hypoalbuminemia is also a recurrent finding in chronic disease, data from predominantly community-acquired sepsis showed that hypoalbuminemia is related to infection and that albumin could act as an independent risk parameter^26,27^. However, similar to lactate levels, serum albumin levels are also affected by multiple conditions including inflammation, malnutrition, and liver cirrhosis. Since each of the two parameters independently predicts mortality, a combination of both seems to be useful in order to further increase the predictive value^28^. The L/A ratio may be a reasonable prognostic marker of sepsis considering that there are many physiological changes that need to be taken into consideration during sepsis; however, the use of the L/A ratio will require further validation prior to clinical application^29–31^. A study by Bou Chebl et al.^32^, showed that the L/A ratio has better prognostic performance than initial serum lactate for in-hospital mortality in adult septic patients.

Given the limitations of lactate and the need for a surrogate marker and considering many physiological changes during sepsis and as the use of the L/A ratio will require further validation prior to clinical application, we conducted this study. The objective of this study was to find a valuable prognostic factor between L/A ratio, serum lactate levels and lactate clearance during early resuscitation of critically ill patients with septic shock. In addition, also the cumulative effect of these markers was evaluated to predict the consequences of septic shock.

## Results

### Baseline characteristics and clinical outcomes

One hundred and fifty one patients with the diagnosis of septic shock were enrolled in this study. Of them 80 (53%) patients were men and 71 (47%) were female. The mean ± SD age of the patients was 67.95 ± 6.58 years. The mean ± SD of APACHE IV and SOFA scores of the patients were 29.50 ± 2.97 and 14.58 ± 1.43, respectively. In terms of outcomes, the incidence of mortality was 33.8%. The mean ± SD of ICU LOS, MV duration and duration of using vasopressor were 11.21 ± 3.22, 6.97 ± 2.67 and 4.91 ± 2.03 days, respectively. Thirty five (23.2%) patients’ needs RRT. Baseline characteristics and clinical outcomes of the patients according to survivors and non-survivors are shown in Table 1. There was no significant difference between survivors and non-survivors in regards of age, gender, suspected infection source, and positive blood culture (*P* > 0.005). The median of APACHE IV (31 vs. 29, *P* < 0.001), SOFA (16 vs. 14, *P* < 0.001), ICU LOS (12 vs. 10, *P* < 0.001), duration of MV (8 vs. 6, *P* < 0.001) and duration of using vasopressor (6 vs. 4, *P* < 0.001) were significantly higher in the non-survivors group than the survivors group. In addition, multiple organ failure (70% vs. 25%, *P* < 0.001) and RRT (35.3% vs. 17%, *P* = 0.012) were significantly higher in the non-survivors group than the survivors group. However, the comorbidities (80.4% vs. 94%, *P* = 0.010) and median of albumin levels were (3.3 vs. 3, *P* < 0.001) significantly lower in the non-survivors group than that in the survivors group.

**Table 1:** Demographic, clinical characteristics and outcomes of participants according to survivor and non-survivor groups.

Variables	Total participants (n = 151)	Survivors (n = 100)	Non-survivors (n = 51)	*P* value
**Age**				
Mean ± SD (Year) (Range)	67.95 ± 6.58 (45–78)	68.62 ± 5.42 (48–78)	66.67 ± 8.29 (45–77)	0.086
Median (IQR)	69 (65–72)	70 (66–72)	69 (62–73)	
**Gender**				
Male (%)	80 (53%)	55 (55)	25 (49)	0.486
Female (%)	71 (47%)	45 (45)	26 (51)	
**Comorbidity**				
Yes (%)	135 (89.4%)	94 (94)	41 (80.4)	0.010*
No (%)	16 (10.6%)	6 (6)	10 (19.6)	
**Type of comorbidities**				
IHD (%)	39 (25.8)	29 (29)	10 (19.6)	0.212
DM (%)	38 (25.2)	22 (22)	16 (31.4)	0.209
HTN (%)	26 (17.2)	16 (16)	10 (19.6)	0.579
CVA (%)	15 (9.9)	10 (10)	5 (9.8)	0.980
CHF (%)	22 (14.6)	17 (17)	5 (9.8)	0.236
HLP (%)	7 (4.6)	4 (4)	3 (5.9)	0.603
Others (%)	2 (1.3)	0	2 (3.9)	0.046*
**Suspected infection source**				
Abdominal (%)	15 (9.9)	10 (10)	5 (9.8)	0.601
Urinary (%)	44 (29.1)	32 (32)	12 (23.5)	
Lung (%)	38 (25.2)	26 (26)	12 (23.5)	
Intravenous catheter (%)	15 (9.9)	10 (10)	5 (9.8)	
CNS (%)	17 (11.3)	9 (9)	8 (15.7)	
Skin (%)	16 (10.6)	10 (10)	6 (11.8)	
Catheter (%)	4 (2.6)	0	1 (2)	
Intravenous line (%)	1 (0.7)	0	1 (2)	
CV line (%)	1 (0.7)	3 (3)	1 (2)	
**Multiple organ failure**				
Yes (%)	60 (40)	25 (25)	35 (70)	< 0.001*
No (%)	90 (60)	75 (75)	15 (30)	
**Blood culture**				
Yes (%)	83 (56.5)	56 (57.1)	27 (55.1)	0.814
No (%)	64 (43.5)	42 (42.9)	22 (44.9)	
**APACHE IV score**				
Mean ± SD (range)	29.50 ± 2.97 (23–38)	28.58 ± 2.67 (23–35)	31.29 ± 2.70 (23–38)	< 0.001*
Median (IQR)	29 (27–32)	29 (27–30.75)	31 (30–33)	
**SOFA score**				
Mean ± SD (Range)	14.58 ± 1.43 (11–18)	14.07 ± 1.19 (11–17)	15.59 ± 1.34 (13–18)	< 0.001*
Median (IQR)	14 (14–16)	14 (13–15)	16 (15–16)	
**ICU length of stay**				
Mean ± SD (Day) (Range)	11.21 ± 3.22 (4–20)	10.41 ± 2.73 (5–18)	12.85 ± 3.56 (4–20)	< 0.001*
Median (IQR)	11 (9–12)	10 (8–12)	12 (10.25–16)	
**Duration of MV**				
Mean ± SD (Day) (Range)	6.97 ± 2.67 (6–16)	5.94 ± 1.93 (2–11)	8.98 ± 2.80 (4–16)	< 0.001*
Median (IQR)	6 (5–8)	6 (5–7)	8 (7–11)	
**Duration of using vasopressor**				
Mean ± SD (Day) (Range)	4.91 ± 2.03 (2–12)	3.92 ± 1.26 (2–8)	6.86 ± 1.83 (4–12)	< 0.001*
Median (IQR)	4 (3–6)	4 (3–5)	6 (6–8)	
**Albumin**				
Median (IQR)	3.3 (3.1–3.4)	3.4 (3.3–3.5)	3.0 (2.9–3.1)	< 0.001*
**RRT**				
Yes (%)	35 (23.2)	17 (17)	18 (35.3)	0.012*
No (%)	116 (76.8)	83 (83)	33 (64.7)	

CHF, congestive heart failure; CVA, cerebral vascular accident; DM, diabetes mellitus; HTN, hypertension; IHD, ischemic heart disease; HLP, hyperlipidemia; MV, mechanical ventilator; APACHE, acute physiology and chronic health evaluation; SOFA, sequential organ failure assessment; RRT, renal replacement therapy.

*P < 0.05 considered as significant.

### Time trend changes of blood-based biomarkers

Time trend changes of lactate and lactate clearance according to survivor and non-survivor groups are presented in Table 2. According to the results, the changes of lactate and lactate clearance based on time and group interaction were significant in adjusted model (P < 0.001). According to the results, the mean level of lactate at 6 (3.17 ± 0.267 vs. 3.35 ± 0.546, *P* = 0.010), 12 (2.58 ± 0.330 vs. 3.05 ± 0.559, *P* < 0.001), and 24 (2.05 ± 0.299 vs. 2.82 ± 0.480, *P* < 0.001) hours after ICU admission was significantly lower in survivors than the non-survivors. However, lactate clearance at 6 (19.39 ± 6.14 vs. 15.05 ± 5.92, *P* < 0.001), 12 (34.43 ± 8.08 vs. 22.14 ± 9.42, *P* < 0.001) and 24 (47.87 ± 7.89 vs. 27.61 ± 7.75, *P* < 0.001) hours after ICU admission was significantly higher in the survivors than the non-survivors.

**Table 2:** Time trend changes of blood-based biomarkers in patients with septic shock according to survivor and non-survivor groups.

Groups	Time trends				Time interaction		Time * group interaction	
	Baseline	6 h	12 h	24 h	Unadjusted (*P* value)^b^	Adjusted (*P* value)^c^	Unadjusted (*P* value)^d^	Adjusted (*P* value)^e^
**Lactate/albumin**								
Survivors	0.149 ± 0.036	–	–	–	–	–	–	–
Non-survivors	0.259 ± 0.053	–	–	–	–	–	–	–
*P* value^a^	< 0.001*							
**Lactate**								
Survivors	3.94 ± 0.248	3.17 ± 0.267	2.58 ± 0.330	2.05 ± 0.299	< 0.001*	0.734	< 0.001*	< 0.001*
Non-survivors	3.93 ± 0.612	3.35 ± 0.546	3.05 ± 0.559	2.82 ± 0.480				
*P* value^a^	0.897	0.010*	< 0.001*	< 0.001*				
**Lactate clearance**								
Survivors	–	19.39 ± 6.14	34.43 ± 8.08	47.87 ± 7.89	< 0.001*	0.327	< 0.001*	< 0.001*
Non-survivors	–	15.05 ± 5.92	22.14 ± 9.42	27.61 ± 7.75				
*P* value^a^		< 0.001*	< 0.001*	< 0.001*				

*P < 0.05 considered as significant.

^a^*P* value based on t-test between groups.

^b^Unadjusted *P* value based on time interaction in repeated measures ANOVA.

^c^Adjusted *P* value (adjusting for age, gender, comorbidities, SOFA and APACHE IV) based on time interaction in repeated measures ANOVA.

^d^Unadjusted *P* value based on time * group interaction in repeated measures ANOVA.

^e^Adjusted *P* value (adjusting for age, gender, comorbidities, SOFA and APACHE IV) based on time * group interaction in repeated measures ANOVA.

### Findings of logistic regression analysis

Univariate and multivariate logistic regression analysis to determine the effect of baseline characteristics and biomarkers on mortality and MV duration are presented in Table 3. Multivariate logistic regression analysis with adjustments for baseline imbalances according to age, gender, comorbidities, APACHE IV, SOFA, multiple organ failure, and blood culture variables revealed that patients with increased lactate clearance levels at 24 h after septic shock recognition had significantly decreased mortality (OR 0.721, 95% CI 0.608–0.854, *P* < 0.001). However, the OR of mortality significantly increased with L/A ratio at 6 h (OR 7.57 × 10^18^, 95% CI 11,049.220–5.19 × 10^23^, *P* = 0.003), and serum lactate level at 24 h (OR 12.621, 95% CI 4.833–14.944, *P* < 0.001). In addition, the risk of mortality was increased by age (OR 1.145, 95% CI 1.007–1.303, *P* = 0.039), having comorbidities (OR 3.821, 95% CI 1.302–11.213, *P* = 0.015), higher score of SOFA (OR 2.674, 95% CI 1.271–5.624, *P* = 0.01), having multiple organ failure (OR 4.179, 95% CI 1.069–8.312, *P* < 0.001), longer ICU LOS (OR 1.634, 95% CI 1.135–4.243, *P* = 0.002), prolong MV duration (OR 1.928, 95% CI 1.105–3.363, *P* = 0.021), and more days of using vasopressor (OR 3.753, 95% CI 2.446–5.759, *P* < 0.001).

**Table 3:** Univariate and multivariate logistic regression analysis to determine the effect of baseline characteristics and biomarkers on mortality and MV duration.

Variables	Univariate		Multivariate	
	OR (95% CI)	*P* value	OR (95% CI)	*P* value
Mortality (yes vs. no)				
Age	0.957 (0.909–1.007)	0.090	1.145 (1.007–1.303)	0.039*
Gender **(female vs. male)**	0.787 (0.4–1.546)	0.487	–	–
Comorbidity **(yes vs. no)**	6.613 (1.512–28.918)	0.012*	3.821 (1.302–11.213)	0.015*
APACHE IV	1.463 (1.253–1.708)	< 0.001*	–	–
SOFA	2.545 (1.813–3.572)	< 0.001*	2.674 (1.271–5.624)	0.01*
Multiple organ failure **(yes vs. no)**	7.000 (3.289–14.9)	< 0.001*	4.179 (1.069–8.312)	< 0.001*
Blood culture **(yes vs. no)**	0.920 (0.461–1.836)	0.814	–	–
ICU length of stay	1.280 (1.135–1.443)	< 0.001*	1.634 (1.135–4.243)	0.002*
Duration of MV	1.733(1.426–2.106)	< 0.001*	1.928 (1.105–3.363)	0.021*
Duration of using vasopressor	4.538 (2.483–8.295)	< 0.001*	3.753 (2.446–5.759)	< 0.001*
RRT **(yes vs. no)**	0.376 (0.173–0.816)	0.013*	–	–
Lactate/Albumin	1.04 × 10^18^ (5.85 × 10^12^–84 × 10^23^)	< 0.001*	7.57 × 10^18^ (11,049.220–5.19 × 10^23^**)**	0.003*
Lactate baseline	0.946 (0.413–2.166)	0.896	–	–
Lactate at 6 h	3.468 (1.312–9.168)	0.012*	4.459 (0.158–125.51)	0.381
Lactate at 12 h	6.375 (4.467–8.963)	< 0.001*	2.786 (0.077–10.678)	0.576
Lactate at 24 h	9.628 (2.767–10.763)	< 0.001*	12.621 (4.833–14.944)	< 0.001*
Clearance lactate at 6 h	0.879 (0.82–0.942)	< 0.001*	0.972 (0.858–1.102)	0.66
Clearance lactate at 12 h	0.857 (0.812–0.905)	< 0.001*	0.964 (0.834–1.114)	0.619
Clearance lactate at 24 h	0.731 (0.655–0.815)	< 0.001*	0.721 (0.608–0.854)	< 0.001*
MV duration (≥ 6 days vs. < 6 days)				
Age	0.976 (0.921–1.033)	0.395	–	–
Gender **(female vs. male)**	0.618 (0.302–1.264)	0.187	–	–
Comorbidity **(yes vs. no)**	7.856 (0.995–10.122)	0.998	–	–
APACHE IV	1.335 (1.158–1.538)	< 0.001*	0.977 (0.533–1.79)	0.940
SOFA	1.883 (1.377–2.575)	< 0.001*	1.255 (1.361–4.367)	0.021*
Multiple organ failure **(yes vs. no)**	11.2 (3.742–33.522)	< 0.001*	0.529 (0.089–3.126)	0.482
Blood culture **(yes vs. no)**	1.546 (0.757–3.161)	0.232	–	–
ICU length of stay	4.005 (2.431–6.6)	< 0.001*	4.547 (2.282–9.061)	< 0.001*
RRT **(yes vs. no)**	5.831 (1.682–20.214)	0.005*	0.371 (0.025–5.511)	0.471
Duration of using vasopressor	4.118 (2.478–6.845)	< 0.001*	2.522 (1.203–5.289)	0.014*
Lactate/Albumin	1.41 × 10^13^ (22,546,848.8–8.90073 × 10^18^)	< 0.001*	6.03 × 10^13^ (1.918 × 10^16^–10.89 × 10^16^)	0.003*
Lactate baseline	1.084 (0.459–2.561)	0.854	0.835 (0.118–5.894)	0.857
Lactate at 6 h	2.018 (0.805–5.056)	0.134	0.248 (0.023–2.676)	0.251
Lactate at 12 h	4.209 (1.718–10.316)	0.002	0.269 (0.019–3.873)	0.335
Lactate at 24 h	10.10 (3.533–28.869)	< 0.001*	18.83 (1.416–25.406)	0.026*
Clearance lactate at 6 h	0.949 (0.897–1.004)	0.070	1.074 (0.978–1.179)	0.136
Clearance lactate at 12 h	0.926 (0.885–0.968)	0.001*	1.041 (0.94–1.153)	0.441
Clearance lactate at 24 h	0.911 (0.875–0.948)	< 0.001*	0.891 (0.804–0.988)	0.029*

*OR* odds ratio, *CI* confidence interval.

*P < 0.05 considered as significant.

Multivariate logistic regression analysis in regards to predict MV duration revealed that patients with increased lactate clearance levels at 24 h after septic shock recognition had significantly lower MV duration less than 6 days (OR 0.891, 95% CI 0.804–0.988, *P* = 0.029). However, the OR of MV duration more than 6 days significantly increased with L/A ratio at 6 h (OR 6.03 × 10^13^, 95% CI 1.918 × 10^16^–10.89 × 10^16^, *P* = 0.003), and serum lactate level at 24 h (OR 18.83, 95% CI 1.416–25.406, *P* = 0.026). In addition, the risk of MV duration more than 6 days was increased by higher score of SOFA (OR 1.255, 95% CI 1.361–4.367, *P* = 0.021), prolong ICU LOS (OR 4.547, 95% CI 2.282–9.061, *P* < 0.001) and more days of using vasopressor (OR 2.522, 95% CI 1.203–5.289, *P* = 0.014). Univariate and multivariate logistic regression analysis to determine the effect of baseline characteristics and biomarkers to predict other outcomes such as ICU length of stay (≥ 11 days vs. < 11 days), RRT (yes vs. no) and duration of using vasopressor (≥ 4 days vs. < 4 days) are available at Supplementary File: Tables 1 and 2.

### Predicting outcomes by biomarkers

Table 4 shows biomarker performance to predict septic shock outcomes with cutoff points. Best performing predictive value for mortality were related to lactate clearance at 24 h, L/A ratio at 6 h and lactate levels at 24 h with (AUC 0.963, 95% CI 0.918–0.987, *P* < 0.001), (AUC 0.917, 95% CI 0.861–0.956, *P* < 0.001), and (AUC 0.904, 95% CI 0.845–0.946, *P* < 0.001), respectively. The AUC value of the L/A ratio at 6 h in all patients was (AUC 0.917, 95% CI 0.861–0.956, *P* < 0.001) and was significantly higher than that of lactate level (AUC 0.657, 95% CI 0.576–0.733, *P* = 0.002) as well as that of lactate clearance level (AUC 0.708, 95% CI 0.627–0.780, *P* < 0.001) at 6 h after septic shock recognition (*P* < 0.001). Best performing predictive value for RRT were related to L/A ratio at 6 h, lactate level at 24 h and lactate clearance at 24 h with (AUC 0.703, 95% CI 0.623–0.775, *P* < 0.001), (AUC 0.696, 95% CI 0.616–0.768, *P* = 0.002), and (AUC 0.671, 95% CI 0.588–0.747, *P* = 0.002), respectively. In regards of prediction ICU LOS, MV duration and also duration of using vasopressor, The L/A ratio has better prognostic performance for RRT than serum lactate and lactate clearance in adult patients with septic shock. Best performing predictive value for ICU LOS were related to L/A ratio at 6 h, lactate clearance at 24 h and lactate at 24 h with (AUC 0.709, 95% CI 0.630–0.780, *P* < 0.001), (AUC 0.684, 95% CI 0.602–0.759, *P* < 0.001), and (AUC 0.675, 95% CI 0.594–0.750, *P* < 0.001), respectively. However, Best performing predictive value for duration of using vasopressor were related to clearance lactate at 24 h (AUC 0.808, 95% CI 0.734–0.869, *P* < 0.001), and the followed by lactate at 24 h (AUC 0.801, 95% CI 0.728–0.862, *P* < 0.001) and L/A ratio at 6 h with (AUC 0.788, 95% CI 0.714–0.850, *P* < 0.001). The ROC curves for all biomarkers to predict outcomes are presented in Supplementary File: Figs. 1–5.

**Table 4:** Performance of blood-based biomarkers in predicting outcomes.

Markers	AUC (95% CI)	*P* value	SN	SP	PPV	NPV	LR+	LR−	Youden Index	Accuracy	Cut-point
**Mortality (yes vs. no)**											
Lactate/albumin	0.917 (0.861–0.956)	< 0.001*	100	63	58	100	2.7	0	0.63	75.49	> 0.1
Lactate baseline	0.527 (0.444–0.609)	0.633	31.3	92.9	69.7	72.4	4.48	0.74	0.24	72.05	> 4.2
Lactate 6 h	0.657 (0.576–0.733)	0.002*	41.18	89.9	68	75	4.08	0.65	0.31	73.40	> 3.5
Lactate 12 h	0.779 (0.704–0.842)	< 0.001*	66.67	83.84	68.22	83.1	4.7	0.40	0.50	78.11	> 2.8
Lactate 24 h	0.904 (0.845–0.946)	< 0.001*	84.31	85.86	75.6	91.4	6.02	0.18	0.70	85.43	> 2.3
Clearance lactate 6 h	0.708 (0.627–0.780)	< 0.001*	62.75	76	57.3	79.8	2.61	0.49	0.37	71.49	≤ 15.8
Clearance lactate 12 h	0.846 (0.776–0.900)	< 0.001*	76.47	86	73.7	87.6	5.46	0.27	0.62	82.76	≤ 27.9
Clearance lactate 24 h	0.963 (0.918–0.987)	< 0.001*	94.12	91	83.3	96.7	10.4	0.06	0.86	92.06	≤ 35.1
**RRT (yes vs. no)**											
Lactate/albumin	0.703 (0.623–0.775)	< 0.001*	82.86	49.14	32.9	90.5	1.63	0.35	0.32	56.95	> 0.1
Lactate baseline	0.646 (0.563–0.722)	0.016*	62.86	63.48	34.18	84.9	1.72	0.59	0.28	63.33	> 4
Lactate 6 h	0.645 (0.562–0.721)	0.019*	62.86	63.79	34.40	85.1	1.74	0.58	0.28	63.58	> 3.2
Lactate 12 h	0.649 (0.567–0.725)	0.007*	55.88	75	39.61	84.4	2.17	0.61	0.31	70.19	> 2.9
Lactate 24 h	0.696 (0.616–0.768)	0.002*	64.71	62.93	33.87	84.8	1.70	0.59	0.27	62.91	> 2.2
Clearance lactate 6 h	0.597 (0.512–0.677)	0.102	50.00	72.97	33.99	82.6	1.70	0.70	0.23	65.56	≤ 15.6
Clearance lactate 12 h	0.614 (0.529–0.693)	0.025*	85.29	44.14	31.25	90.6	1.48	0.34	0.29	52.44	≤ 34.4
Clearance lactate 24 h	0.671 (0.588–0.747)	0.002*	52.94	79.28	40.79	84.4	2.25	0.60	0.32	70.80	≤ 31.9
**ICU length of stay (≥ 11 days vs. < 11 days)**											
Lactate/Albumin	0.709 (0.630–0.780)	< 0.001*	76.92	61.64	68.19	71.4	2.01	0.37	0.38	69.54	> 0.1
Lactate baseline	0.522 (0.439–0.604)	0.644	85.90	1.39	48.56	15.3	0.88	5.15	0.12	45.70	> 3.4
Lactate 6 h	0.569 (0.486–0.649)	0.141	43.59	73.61	64.15	55.1	1.67	0.76	0.17	58.28	> 3.3
Lactate 12 h	0.593 (0.510–0.672)	0.046*	43.59	77.78	68.00	56.4	1.99	0.72	0.21	60.26	> 2.8
Lactate 24 h	0.675 (0.594–0.750)	< 0.001*	52.56	77.78	71.97	60.6	2.40	0.61	0.30	64.89	> 2.3
Clearance lactate 6 h	0.585 (0.500–0.666)	0.074	42.47	73.61	64.33	55.7	1.68	0.74	0.16	58.93	≤ 15.8
Clearance lactate 12 h	0.625 (0.541–0.704)	0.008*	49.32	75.00	68.37	59.3	2.02	0.64	0.24	62.90	≤ 29.2
Clearance lactate 24 h	0.684 (0.602–0.759)	< 0.001*	58.90	80.56	77.22	65.8	2.99	0.48	0.39	70.19	≤ 38.1
**Duration MV (≥ 6 days vs. < 6 days)**											
Lactate/Albumin	0.754 (0.678–0.821)	< 0.001*	71.03	72.73	86.36	50.7	2.60	0.4	0.43	71.52	> 0.1
Lactate baseline	0.548 (0.465–0.629)	0.318	47.17	68.18	78.28	34.6	1.48	0.77	0.15	53.29	> 4
Lactate 6 h	0.602 (0.519–0.681)	0.030*	26.42	93.18	90.32	34.2	3.84	0.79	0.19	45.70	> 3.5
Lactate 12 h	0.682 (0.601–0.755)	< 0.001*	43.40	90.91	92.00	39.6	4.73	0.63	0.34	56.95	> 2.8
Lactate 24 h	0.764 (0.687–0.829)	< 0.001*	57.55	90.91	93.85	46.5	6.27	0.47	0.48	66.89	> 2.2
Clearance lactate 6 h	0.606 (0.522–0.686)	0.037*	50.47	72.73	81.82	37.6	1.85	0.68	0.20	56.95	≤ 16.2
Clearance lactate 12 h	0.700 (0.618–0.773)	< 0.001*	47.52	86.36	90.00	41.7	3.70	0.57	0.33	60.93	≤ 29.2
Clearance lactate 24 h	0.757 (0.679–0.824)	< 0.001*	61.39	88.64	93.15	50.0	5.59	0.41	0.50	70.86	≤ 41
**Duration of using vasopressor (≥ 4 days vs. < 4 days)**											
Lactate/Albumin	0.788 (0.714–0.850)	< 0.001*	72.90	77.27	88.64	53.9	3.21	0.35	0.50	74.17	> 0.1
Lactate baseline	0.537 (0.453–0.618)	0.445	19.81	95.45	91.30	32.8	4.32	0.84	0.14	41.72	> 4.2
Lactate 6 h	0.655 (0.573–0.731)	0.007*	67.92	59.09	79.69	35.6	1.62	0.74	0.27	54.30	> 3.1
Lactate 12 h	0.749 (0.672–0.816)	< 0.001*	45.28	95.45	96.00	41.6	9.87	0.56	0.40	59.60	> 2.8
Lactate 24 h	0.801 (0.728–0.862)	< 0.001*	52.83	97.73	98.25	45.7	23.1	0.49	0.50	65.56	> 2.3
Clearance lactate 6 h	0.678 (0.596–0.753)	< 0.001*	50.50	79.55	86.36	41.2	2.60	0.59	0.30	60.93	≤ 16.2
Clearance lactate 12 h	0.793 (0.718–0.856)	< 0.001*	72.28	72.73	86.81	53.3	2.71	0.36	0.45	73.51	≤ 34
Clearance lactate 24 h	0.808 (0.734–0.869)	< 0.001*	57.43	97.73	98.46	50.0	26.3	0.41	0.55	70.86	≤ 39.5

CI, confidence interval, SN, Sensitivity; SP, Specificity; LR+, Positive Likelihood Ratio; LR−, Negative Likelihood Ratio; PPV, Positive Predictive value; NPV, Negative Predictive value.

*P* < 0.05 considered as significant.

### Cumulative effect of biomarkers

To have a cumulative effect of markers, we conducted a logistic regression and then using the probability for creating new variables from combined the biomarkers at different time intervals to predict the consequences of septic shock. The cumulative effect of biomarkers on outcomes are presented in Table 5. In combined biomarkers to predict mortality, the highest AUC was related to the combined marker at 24 h (lactate + lactate clearance at 24 h) with (AUC 0.962, 95% CI 0.916–0.987, *P* < 0.001) and the used cutoff value had a value of sensitivity 93.6%, specificity 92.8%, (LR+) 13.11, (LR−) 0.06 and 0.86% of Yuden index. And the followed by the combined marker at 6 h (L/A ratio 6 h + lactate 6 h + lactate clearance 6 h) with (AUC 0.938, 95% CI 0.885–0.971, *P* < 0.001) and the used cutoff value had a value of sensitivity 91.5%, specificity 84.7%, (LR+) 5.98, (LR−) 0.10 and 0.76% of Yuden index. Combined marker at 12 h (lactate + lactate clearance at 12 h) to predict mortality had (AUC 0.846, 95% CI 0.777–0.901, *P* < 0.001) and the used cutoff value had a value of sensitivity 72.3%, specificity 89.8%, (LR+) 5.98, (LR−) 7.09 and 0.31% of Yuden index. No significant differences were found between the two AUCs in combined markers at 6 h and 24 h (0.938 vs. 0.962, *P* = 0.291). However, the AUC in the combined marker at 12 h was significantly lower than the two combined markers at 6 h (0.846 vs. 0.938, *P* = 0.006) and 24 h (0.846 vs. 0.962, *P* = 0.0003).

**Table 5:** Binary logistic regression model to combine biomarkers to predicting outcomes.

Markers	AUC (95% CI)	*P* value	SN	SP	LR+	LR−	YI	Cut-point
**Mortality (yes vs. no)**								
6 h	0.938 (0.885–0.971)	< 0.001*	91.49	84.69	5.98	0.10	0.76	> 0.306703531
12 h	0.846 (0.777–0.901)	< 0.001*	72.34	89.80	7.09	0.31	0.62	> 0.358627361
24 h	0.962 (0.916–0.987)	< 0.001*	93.62	92.86	13.11	0.069	0.86	> 0.442224806
**RRT (yes vs. no)**								
6 h	0.747 (0.668–0.815)	< 0.001*	70.59	75.68	2.90	0.39	0.46	> 0.248886836
12 h	0.688 (0.606–0.762)	0.0002*	50.00	86.49	3.70	0.58	0.36	> 0.290158373
24 h	0.695 (0.613–0.768)	0.0003*	58.82	72.07	2.11	0.57	0.31	> 0.220166037
**ICU length of stay (≥ 11 days vs. < 11 days)**								
6 h	0.693 (0.611–0.766)	< 0.001*	76.71	62.50	2.05	0.37	0.39	> 0.361215256
12 h	0.626 (0.542–0.705)	0.006*	43.84	79.17	1.10	0.71	0.23	> 0.527501309
24 h	0.684 (0.602–0.759)	< 0.001*	58.90	80.56	3.03	0.51	0.39	> 0.540208132
**MV duration (≥ 6 days vs. < 6 days)**								
6 h	0.749 (0.670–0.817)	< 0.001*	68.32	77.27	3.01	0.41	0.45	> 0.797995991
12 h	0.697 (0.615–0.771)	< 0.001*	55.45	81.82	3.05	0.54	0.37	> 0.713307883
24 h	0.758 (0.680–0.825)	< 0.001*	60.40	90.91	6.64	0.46	0.51	> 0.735624058
**Duration of using vasopressor (≥ 4 days vs. < 4 days)**								
6 h	0.807 (0.733–0.868)	< 0.001*	63.37	86.36	4.65	0.42	0.49	> 0.820505663
12 h	0.792 (0.717–0.855)	< 0.001*	62.38	84.09	3.92	0.45	0.46	> 0.726994118
24 h	0.807 (0.733–0.867)	< 0.001*	58.42	97.73	25.70	0.43	0.56	> 0.787125813

*P* < 0.05 considered as significant.

SN, sensitivity; SP, specificity; LR+, positive likelihood ratio; LR−, negative likelihood ratio.

Best performing predictive value for RRT and ICU LOS were related to combined markers at 6 h with (AUC 0.747, 95% CI 0.668–0.815, *P* < 0.001) and the used cutoff value had a value of sensitivity 70.6%, specificity 76%, (LR+) 2.90, (LR−) 0.39 and 0.46% of Yuden index, and (AUC 0.693, 95% CI 0.611–0.766, *P* < 0.001) and the used cutoff value had a value of sensitivity 76.7%, specificity 62.5%, (LR+) 2.05, (LR−) 0.37 and 0.39% of Yuden index, respectively. No significant differences were found between the AUCs in combined markers at 6 h, 12 h and 24 h for predicting RRT. However, the AUC in the combined marker at 12 h for predicting ICU LOS was significantly lower than the combined marker at 24 h (0.624 vs. 0.684, *P* = 0.036).

Best performing predictive value for MV duration was related to combined markers at 24 h with (AUC 0.758, 95% CI 0.680–0.825, *P* < 0.001) and the used cutoff value had a value of sensitivity 60.4%, specificity 90.9%, (LR+) 6.64, (LR−) 0.46 and 0.51% of Yuden index. Significant difference was found between AUCs in combined markers at 12 h and 24 h (0.697 vs. 0.758, *P* = 0.022). In terms of predicting the duration of use of vasopressors, all combined markers had the same power and no significant differences were observed between them.

### Results of comparison of AUCs

**Figure 1:**
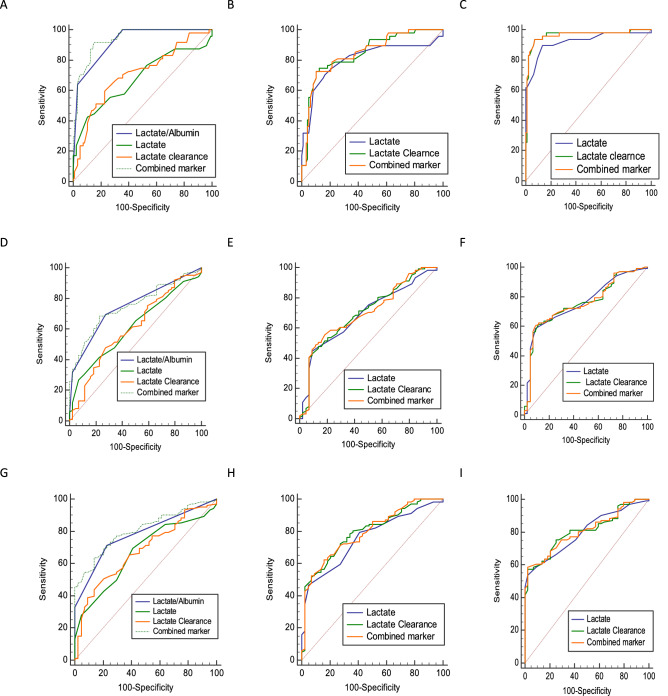
Comparison of ROC curves of single markers with combine marker to predict mortality (**A**–**C**), MV duration (**D**–**F**), and duration of using vasopressors at 6 h, 12 h and 24 h, respectively.

Comparison of AUCs was performed between individual biomarkers and combined markers at different time interval using DeLong test and the results for predicting mortality, MV duration and duration of using vasopressors are shown in Fig. 1. To predicting mortality, combined marker at 6 h and also single marker of L/A ratio had a best performing and were comparable with single marker of lactate and lactate clearance (*P* < 0.001). However, a combination of L/A ratio + lactate at 6 h + lactate clearance at 6 h as the combined marker at 6 h did not improve diagnostic accuracy over the single marker of L/A ratio (0.938 vs. 0.921, *P* = 0.137), (Fig. 1A). A combination of lactate at 12 h + lactate clearance at 12 h as the combined marker at 12 h and also combination of lactate at 24 h + lactate clearance at 24 h as the combined marker at 24 h to predict mortality did not improve diagnostic accuracy over the single marker of lactate at 12 h (0.846 vs. 0.804, *P* = 0.238), lactate clearance at 12 h (0.846 vs. 0.846, *P* = 0.962), and lactate at 24 h (0.962 vs. 0.922, *P* = 0.077), lactate clearance at 24 h (0.962 vs. 0.963, *P* = 0.693), respectively (Fig. 1B,C).

Best performing predictive value for MV duration was related to combined marker at 6 h, which did not improve diagnostic accuracy over the single marker of L/A ratio (0.749 vs. 0.746, *P* = 0.888). However, diagnostic accuracy of combined marker at 6 h was significantly higher than single marker of lactate at 6 h (0.749 vs. 0.614, *P* = 0.001) and clearance lactate at 6 h (0.749 vs. 0.606, *P* = 0.0005) (Fig. 1D). A combined markers at 12 h and 24 h to predict MV duration did not improve diagnostic accuracy over the single markers of lactate and lactate clearance at (*P* < 0.05) (Fig. 1E,F). Best performing predictive value for duration of using vasopressors was related to combined marker at 6 h, which did not improve diagnostic accuracy over the single marker of L/A ratio (0.807 vs. 0.780, *P* = 0.135). However, diagnostic accuracy of combined marker at 6 h was significantly higher than single marker of lactate at 6 h (0.807 vs. 0.668, *P* = 0.001) and clearance lactate at 6 h (0.807 vs. 0.678, *P* = 0.0006) (Fig. 1G). A combined markers at 12 h and 24 h to predict duration of using vasopressors did not improve diagnostic accuracy over the single markers of lactate and lactate clearance (*P* < 0.05) (Fig. 1H,I). The results of pairwise comparison of ROC curves to predict other outcomes are presented in (Supplementary File: Fig. 6–20).

## Discussion

Critically ill patients with severe sepsis or septic shock have a poor prognosis and are associated with a high economic burden. Therefore, predicting the outcomes of these patients can help clinicians make relevant clinical decisions. In this bi-center observational study, we compared the predictive ability of the L/A ratio, lactate and lactate clearance biomarkers on the outcomes of patients with septic shock during initial resuscitation. The results showed that the lactate clearance at 24 h had better prognostic performance for mortality and duration of using vasopressor. However, the L/A ratio had better prognostic performance than serum lactate and lactate clearance for RRT, ICU LOS and MV duration. The AUC of the L/A ratio at 6 h for predicting the outcomes of septic shock was better than that of lactate and lactate clearance levels at 6 h later from septic shock recognition. The AUC of lactate and lactate clearance increased with time and our findings showed that 24 h from septic shock recognition was the optimal timing of these biomarkers for predicting outcomes. In addition, to have the cumulative effect of biomarkers, we combined the biomarkers based on their measurement time to predict the consequences of septic shock. The combined model form biomarkers had a higher-precision AUCs, SN, SP, LR+, and lower LR− values for predicting outcomes than models developed from single markers of lactate and lactate clearance. However, a combined markers at 6 h to predict outcomes did not improve diagnostic accuracy over the single markers of L/A ratio.

Previous studies consistent with this study have shown that the lactate and lactate clearance could predict the survival outcome in patients with severe sepsis or septic shock^9,20,33^. However, lactate levels are also affected by other factors; for instance, liver or kidney dysfunction may lead to abnormal lactate due to clearance disorders. Albumin and lactate each independently predicts mortality, and the values inversely change by different mechanisms^25,34^. However, a comprehensive combination of both parameters seems to increase the predictive value. The L/A ratio reflects the reverse changes caused by two different mechanisms and is thus more accurate in predicting the outcome in critically ill patients with septic shock. Therefore, the use of L/A ratio as marker has been suggested in many studies. Shin et al.^35^, evaluated the value of the L/A ratio in predicting the outcomes of patients with severe sepsis. The results showed that the AUC of the L/A ratio was higher than that for lactate alone. Further, in patients with lactate clearance disorder, the AUC of the L/A ratio was still higher than that of lactate. Wang et al.^36^, evaluated the predictive role of the L/A ratio regarding the risk of multiple organ dysfunction syndromes (MODS) and mortality among patients with severe sepsis or septic shock and the results showed that the L/A ratio correlated with the risk of MODS and death. A study by Lichtenauer et al.^28^, showed that the L/A ratio contributes to the risk stratification for patients with sepsis. Cakir et al.^37^, in their study consisting of 1,136 septic patients showed that L/A ratio has better predictive validity compared to lactate or albumin alone in septic patients.

Contrary to our study, a study by Mustafa et al.^38^, showed that L/A ratio is a better discriminator of MODS development and mortality than lactate clearance in pediatric patients with severe sepsis or septic shock. It should be noted that the ratio of lactate to albumin in the study was collected 6 h after the initial diagnosis of septic shock in patients. And when we compared the predictive ability of the L/A ratio with lactate and lactate clearance at 6 h, L/A ratio had better prognostic performance for mortality. Furthermore, differences between the results of our study and the previous one can be due to the lower sample size of the mentioned study (Mustafa et al.), better compliance of our staff regarding performing SCCM resuscitation bundles especially regarding lactate, the difference in the population enrolled, and finally the difference in management strategy of the two departments (emergency medicine vs. critical care medicine). Considering that lactate is primarily metabolized by the liver and to a lesser extent by the kidney, these patients often present to the emergency department with elevated lactate levels, but we excluded this population in our study. Because of impaired lactate clearance in patients with liver failure, physicians are usually confused with the permanent high lactate levels which usually cause them to stop fluid administration.

According to our results and previous studies, at the same lactate level, using the L/A ratio can further identify high-risk patients^32,35^. Therefore, monitoring the L/A ratio may help to manage critically ill patients with septic shock better in clinical practice. Different studies showed different cut-off values for Lactate/Alb ratio regarding mortality in septic patients^32,35^. The optimal cut-off value of the Lactate/Alb ratio remains unknown and the authors recommend the need for a future study that is prospective in nature to help establish the optimal Lactate/Alb ratio. There were several limitations to this study. First, our participants was critically ill patients with various pre-existing comorbidities which acute dysfunction precludes a correct interpretation of results. Second, we excluded patients with advanced liver dysfunction and ARDS but cannot rule-out some degree of subclinical dysfunction. Third, we did not measure albumin at fist hours of diagnosis and it can be considered as one of limitation of this study. However, it seems that the change in albumin during first 6 h would not be significant but the change in lactate will be dependent on the perfusion and can be significant during the first 6 h and this is the rational for using a lactate/albumin ratio as a diagnostic tool in critically ill patients with sepsis.

In summary, the predictive ability of both lactate and lactate clearance increased over time, which 24 h from septic shock recognition was the optimal timing of these biomarkers for predicting outcomes. Our study supports the existing literature on the use of L/A ratio as a prognostic marker in patients with sepsis with fair discriminative value. The AUC of the L/A ratio to predict septic shock outcomes was better than the levels of lactate and lactate clearance at 6 h after septic shock diagnosis. The L/A ratio is very easy to obtain and therefore may be very useful for classifying risk in patients with septic shock but it requires further validation prior to clinical application. However, determining a valid cut off point during definite times like 3 or 6 h after resuscitation can show important data regarding the quality of resuscitation and outcome of patients.

## Methods

### Study design and setting

This was a multi-center observational study which conducted in the two Intensive Care Units (ICUs) at Shohada and Imam Reza Hospitals, Tabriz, Iran, between Sept 2018 and Jan 2021. The aim of this study was to evaluate the prognostic value of the L/A ratio compared to that of lactate and lactate clearance in predicting outcomes in patients with septic shock. The protocol study was reviewed and approved by Research Ethics Committees of Islamic Azad University-Tabriz Branch (IR.TBZMED.REC.1397.1014). Informed consent was obtained from all subjects and/or their legal guardian(s). This observational study was conducted and reported in accordance with the recommendations of the Strengthening the Reporting of Observational Studies in Epidemiology (STROBE) statement^39^. In addition, the study was conducted in accordance with the Declaration of Helsinki and subsequent^40^.

### Study population

All adult patients (≥ 18 years) with septic shock admitted at ICUs were considered as eligible for this study. Sepsis was defined according to the Third International Consensus Definitions for Sepsis and Septic Shock (Sepsis-3) guidelines as the presence of an infection with signs of organ dysfunction, which are represented by a Sequential [Sepsis-related] Organ Failure Assessment (SOFA) score of two points or greater. Septic shock was defined as a vasopressor requirement to maintain a mean arterial pressure (MAP) of 65 mm Hg or greater, and a serum lactate level > 2 mmol/L (> 18 mg/dL) in the absence of hypovolemia^41^. Patients who met the criteria of sepsis-3 were included in the study. Exclusion criteria included patients who were identified as septic patients but did not meet sepsis-3 criteria, patients who did not have "resuscitation" status, patients with previous history of septic shock during past 3 months, patients with advanced chronic obstructive pulmonary disease (COPD), patients with liver failure and those who were transferred to another hospital during initial resuscitation.

### Treatment procedure

All patients with septic shock were treated according to the standard protocol recommended by the Surviving Sepsis Campaign (SSC) guidelines, including initial crystalloid bolus infusions, blood culture, broad-spectrum antibiotics, vasopressors, and lung-protective strategy ventilation^42^. According to the surviving sepsis campaign, blood culture, empirical antibiotics, and initial lactate measurement were conducted within 3 h from shock recognition fluid resuscitation; also, re estimation of the volume status and lactate re measurement were done promptly within 6 h of vasopressor use^42^.

### Data collection

Demographic and clinical data, including age, gender, comorbidities, suspected infection source, organ failure, and blood culture were collected for each participants. The Sequential Organ Failure Assessment (SOFA) score and acute physiology and chronic health evaluation (APACHE IV) were calculated at the ICU admission^43,44^.

Initial serum lactate levels were measured at shock recognition and we followed the lactate level at 6, 12 and 24 h from initial measurement. Albumin levels was measured at 6 h after shock recognition. However, this initial 6 h resuscitation period was divided by two 3 h' time; The 3-h recommendations, which must be carried out within 3 h from the first time sepsis is suspected, includes: (a) obtain a blood culture before antibiotics, (b) obtain a lactate level, (c) administer broad-spectrum antibiotics, and (d) administer 30 mL/kg of crystalloid fluid for hypotension and the resuscitation bundle is a combination of evidence-based objectives that must be completed within 6 h for patients presenting with severe sepsis, septic shock, and/or lactate > 4 mmol/L (36 mg/dL). Arterial lactate level was analyzed by the “GEM Premier 3500 in Instrumentation Laboratory” automated blood gas analysis system (Werfen, Bedford, MA) during study period. The lactate clearance rate was defined by the equation [(lactate initial − lactate delayed)/lactate initial] × 100%^45^. Lactate clearance levels were measured at 6, 12 and 24 h from shock recognition.

The primary clinical outcome of this study was the mortality rate. Secondary outcomes including ICU length of stay (LOS), need of renal replacement therapy (RRT), duration of mechanical ventilation (MV), and duration of using vasopressor were registered for all participants. We also categorized the quantitative outcomes based on their average as follows: ICU length of stay (≥ 11 days vs. < 11 days), MV duration (≥ 6 days vs. < 6 days), and duration of using vasopressor (≥ 4 days vs. < 4 days).

### Statistical analysis

Power calculations based on main outcome (predictive valuable of biomarkers for mortality) using PASS sample size software version 20.0.6 according type I error 5% determined that 151 patients with septic shock needed to achieve a 95% confidence level and a 90% power^46,47^. All studied variables were tested for normal distribution with Kolmogorov–Smirnov test. Categorical variables are presented as frequency with percentages and continuous variables are presented as mean ± standard deviation (SD) and/or median (IQR). Changes of lactate and lactate clearance biomarkers according to time trends (baseline, 6 h, 12 h and 24 h) were assessed based on two way analysis of variance with repeated measures (RMANOVA), adjusted and non-adjusted models. Adjusted RMANOVA model was done according to the baseline characteristics including age, gender, comorbidities, APACHE IV and SOFA score. The effect of demographic, clinical biomarkers variables on outcomes (mortality, RRT, ICU length of stay, MV duration, and duration of using vasopressor) were determined by univariate and multivariate (to adjust for potential confounders) logistic regression and the results were expressed as odds ratio (OR) with 95% confidence interval (CI).

The receiver operative characteristic (ROC) curve was generated for all biomarkers (L/A ratio, lactate and lactate clearance) at different times. Area under the curve (AUC) figures were calculated alongside sensitivity (SN), specificity (SP), positive predictive value (PPV), negative predictive value (NPV), positive likelihood ratio (LR+), negative likelihood ratio (LR−), Youden Index and accuracy for each biomarkers according to predict each outcomes (primary and secondary). In addition, we combined the markers to create new markers to predict outcomes using binary logistic regression to get the probability and then run a ROC curve using the probability as the test markers and AUCs were compared using the DeLong test. According to general guide for the discriminative power of a test based on ROC, AUC between (0.9–1.0), (0.8–0.9), (0.7–0.8), and (0.6–0.7) was considered as excellent, good, fair, and poor, respectively. Statistical analysis was carried out using SPSS software (ver.21) (SPSS Inc. IL, Chicago, USA) and MedCalc (https://www.medcalc.org/calc/diagnostic_test.php). In all analyses, *P* values less than 0.05 were considered as significant.

### Ethical approval

The protocol study was reviewed and approved by the Research Ethics Committees of Islamic Azad University-Tabriz Branch (IR.TBZMED.REC.1397.1014). This observational study was conducted and reported in accordance with the recommendations of the Strengthening the Reporting of Observational Studies in Epidemiology (STROBE) statement. Informed consent was obtained from all subjects and/or their legal guardian(s). The study was conducted in accordance with the Declaration of Helsinki and subsequent.

## Supplementary Information

Supplementary Information.

## Data Availability

All data collected and analyzed during the current study are available from the corresponding author on reasonable request.

## References

[1] Fleischmann C, Scherag A, Adhikari NK, Hartog CS, Tsaganos T, Schlattmann P (2016). Assessment of global incidence and mortality of hospital-treated sepsis. Current estimates and limitations. Am. J. Respir. Crit. Care Med..

[2] Hamishehkar H, Beigmohammadi MT, Abdollahi M, Ahmadi A, Mahmoodpour A, Mirjalili MR (2010). Identification of enhanced cytokine generation following sepsis. Dream of magic bullet for mortality prediction and therapeutic evaluation. Daru.

[3] Yealy DM, Kellum JA, Huang DT, Barnato AE, Weissfeld LA, Pike F (2014). A randomized trial of protocol-based care for early septic shock. N. Engl. J. Med..

[4] Beck V, Chateau D, Bryson GL, Pisipati A, Zanotti S, Parrillo JE (2014). Timing of vasopressor initiation and mortality in septic shock: A cohort study. Crit. Care.

[5] Gaieski DF, Mikkelsen ME, Band RA, Pines JM, Massone R, Furia FF (2010). Impact of time to antibiotics on survival in patients with severe sepsis or septic shock in whom early goal-directed therapy was initiated in the emergency department. Crit. Care Med..

[6] Samraj RS, Zingarelli B, Wong HR (2013). Role of biomarkers in sepsis care. Shock.

[7] Reinhart K, Bauer M, Riedemann NC, Hartog CS (2012). New approaches to sepsis: Molecular diagnostics and biomarkers. Clin. Microbiol. Rev..

[8] Park IH, Yang JH, Jang WJ, Chun WJ, Oh JH, Park YH (2021). Clinical significance of lactate clearance in patients with cardiogenic shock: Results from the RESCUE registry. J. Intensive Care.

[9] Slottosch I, Liakopoulos O, Kuhn E, Scherner M, Deppe AC, Sabashnikov A (2017). Lactate and lactate clearance as valuable tool to evaluate ECMO therapy in cardiogenic shock. J. Crit. Care.

[10] Bruno RR, Wernly B, Flaatten H, Fjølner J, Artigas A, Bollen Pinto B (2021). Lactate is associated with mortality in very old intensive care patients suffering from COVID-19: Results from an international observational study of 2860 patients. Ann. Intensive Care.

[11] Levy MM, Evans LE, Rhodes A (2018). The surviving sepsis campaign bundle: 2018 update. Intensive Care Med..

[12] Smith ZR, Horng M, Rech MA (2019). Medication-induced hyperlactatemia and lactic acidosis: A systematic review of the literature. Pharmacotherapy.

[13] Scale T, Harvey JN (2011). Diabetes, metformin and lactic acidosis. Clin. Endocrinol..

[14] Friedenberg AS, Brandoff DE, Schiffman FJ (2007). Type B lactic acidosis as a severe metabolic complication in lymphoma and leukemia: A case series from a single institution and literature review. Medicine.

[15] Gabow PA, Clay K, Sullivan JB, Lepoff R (1986). Organic acids in ethylene glycol intoxication. Ann. Intern. Med..

[16] Sterling SA, Puskarich MA, Jones AE (2015). The effect of liver disease on lactate normalization in severe sepsis and septic shock: A cohort study. Clin. Exp. Emerg. Med..

[17] Shin TG, Jo IJ, Hwang SY, Jeon K, Suh GY, Choe E (2016). Comprehensive interpretation of central venous oxygen saturation and blood lactate levels during resuscitation of patients with severe sepsis and septic shock in the emergency department. Shock.

[18] Mahmoodpoor A, Shadvar K, Sanaie S, Golzari SEJ, Parthvi R, Hamishehkar H (2020). Arterial vs venous lactate: Correlation and predictive value of mortality of patients with sepsis during early resuscitation phase. J. Crit. Care.

[19] Nguyen HB, Rivers EP, Knoblich BP, Jacobsen G, Muzzin A, Ressler JA (2004). Early lactate clearance is associated with improved outcome in severe sepsis and septic shock. Crit. Care Med..

[20] Ryoo SM, Lee J, Lee YS, Lee JH, Lim KS, Huh JW (2018). Lactate level versus lactate clearance for predicting mortality in patients with septic shock defined by sepsis-3. Crit. Care Med..

[21] Marty P, Roquilly A, Vallée F, Luzi A, Ferré F, Fourcade O (2013). Lactate clearance for death prediction in severe sepsis or septic shock patients during the first 24 hours in Intensive Care Unit: An observational study. Ann. Intensive Care.

[22] Mahmoodpoor A, Shadvar K, Saghaleini SH, Koleini E, Hamishehkar H, Ostadi Z (2018). Which one is a better predictor of ICU mortality in septic patients? Comparison between serial serum lactate concentrations and its removal rate. J. Crit. Care.

[23] Mahmoodpoor, A., Sanaie, S. & Nader, N. Correlation between arterial and venous lactate levels during initial resuscitation of sepsis. *Original Investigation Posters at Chest Annual Meeting* (2019).

[24] Jones AE, Shapiro NI, Trzeciak S, Arnold RC, Claremont HA, Kline JA (2010). Lactate clearance vs central venous oxygen saturation as goals of early sepsis therapy: A randomized clinical trial. JAMA.

[25] Yin M, Si L, Qin W, Li C, Zhang J, Yang H (2018). Predictive value of serum albumin level for the prognosis of severe sepsis without exogenous human albumin administration: A prospective cohort study. J. Intensive Care Med..

[26] Artero A, Zaragoza R, Camarena JJ, Sancho S, González R, Nogueira JM (2010). Prognostic factors of mortality in patients with community-acquired bloodstream infection with severe sepsis and septic shock. J. Crit. Care.

[27] Magnussen B, Oren Gradel K, Gorm Jensen T, Kolmos HJ, Pedersen C, Just Vinholt P (2016). Association between hypoalbuminaemia and mortality in patients with community-acquired bacteraemia is primarily related to acute disorders. PLoS ONE.

[28] Lichtenauer M, Wernly B, Ohnewein B, Franz M, Kabisch B, Muessig J (2017). The lactate/albumin ratio: A valuable tool for risk stratification in septic patients admitted to ICU. Int. J. Mol. Sci..

[29] Aboraya KM, Makram EF, Ibrahim M, Abdel Rahman A (2021). Serum lactate/albumin ratio as a predictor of morbidity and mortality in patients with severe sepsis and septic shock. Benha Med. J..

[30] Bou Chebl R, Geha M, Assaf M, Kattouf N, Haidar S, Abdeldaem K (2021). The prognostic value of the lactate/albumin ratio for predicting mortality in septic patients presenting to the emergency department: A prospective study. Ann. Med..

[31] Zhu X, Xue J, Liu Z, Dai W, Xu H, Zhou Q (2021). The lactate/albumin ratio predicts mortality in critically ill patients with acute kidney injury: An observational multicenter study on the eICU database. Int. J. Gen. Med..

[32] Bou Chebl R, Jamali S, Sabra M, Safa R, Berbari I, Shami A (2020). Lactate/albumin ratio as a predictor of in-hospital mortality in septic patients presenting to the emergency department. Front. Med..

[33] Takahashi N, Nakada T-A, Walley KR, Russell JA (2021). Significance of lactate clearance in septic shock patients with high bilirubin levels. Sci. Rep..

[34] Bakker J, Nijsten MW, Jansen TC (2013). Clinical use of lactate monitoring in critically ill patients. Ann. Intensive Care.

[35] Shin J, Hwang SY, Jo IJ, Kim WY, Ryoo SM, Kang GH (2018). Prognostic value of the lactate/albumin ratio for predicting 28-day mortality in critically ILL sepsis patients. Shock.

[36] Wang B, Chen G, Cao Y, Xue J, Li J, Wu Y (2015). Correlation of lactate/albumin ratio level to organ failure and mortality in severe sepsis and septic shock. J. Crit. Care.

[37] Cakir E, Turan IO (2021). Lactate/albumin ratio is more effective than lactate or albumin alone in predicting clinical outcomes in intensive care patients with sepsis. Scand. J. Clin. Lab. Invest..

[38] Moustafa AA, Antonios MA, Abdellatif EM, Hussain AH (2018). Association of lactate/albumin ratio level to organ failure and mortality in severe sepsis in a pediatric intensive care unit in Egypt. Turk. J. Pediatr..

[39] von Elm E, Altman DG, Egger M, Pocock SJ, Gøtzsche PC, Vandenbroucke JP (2008). The strengthening the reporting of observational studies in epidemiology (STROBE) statement: Guidelines for reporting observational studies. J. Clin. Epidemiol..

[40] World Medical Association (2013). World Medical Association Declaration of Helsinki: Ethical principles for medical research involving human subjects. JAMA.

[41] Singer M, Deutschman CS, Seymour CW, Shankar-Hari M, Annane D, Bauer M (2016). The third international consensus definitions for sepsis and septic shock (sepsis-3). JAMA.

[42] Rhodes A, Evans LE, Alhazzani W, Levy MM, Antonelli M, Ferrer R (2017). Surviving sepsis campaign: International guidelines for management of sepsis and septic shock: 2016. Intensive Care Med..

[43] Knaus WA, Draper EA, Wagner DP, Zimmerman JE (1985). APACHE II: A severity of disease classification system. Crit. Care Med..

[44] Moreno R, Vincent JL, Matos R, Mendonça A, Cantraine F, Thijs L (1999). The use of maximum SOFA score to quantify organ dysfunction/failure in intensive care. Results of a prospective, multicentre study. Working group on sepsis related problems of the ESICM. Intensive Care Med..

[45] Odom SR, Howell MD, Silva GS, Nielsen VM, Gupta A, Shapiro NI (2013). Lactate clearance as a predictor of mortality in trauma patients. J. Trauma Acute Care Surg..

[46] Hanley JA, McNeil BJ (1983). A method of comparing the areas under receiver operating characteristic curves derived from the same cases. Radiology.

[47] Obuchowski NA, McClish DK (1997). Sample size determination for diagnostic accuracy studies involving binormal ROC curve indices. Stat. Med..

