# Supplementary material for: Comparison of lactate/albumin ratio to lactate and lactate clearance for predicting outcomes in patients with septic shock admitted to intensive care unit: an observational study
Source: Sci Rep. 2022 Jul 29;12:13047. doi: 10.1038/s41598-022-14764-z (PMC9338032; doi:10.1038/s41598-022-14764-z)
Supplement: Supplementary file 1 — Supplementary Information. [file 41598_2022_14764_MOESM1_ESM.docx]

**Comparison of lactate/albumin ratio to lactate and lactate clearance for predicting outcomes in patients with septic shock admitted to intensive care unit: an observational study**

**Author names and affiliations**

**1. Kamran Shadvar:** Department of Anesthesiology and intensive care medicine, Faculty of Medicine, Tabriz University of Medical Sciences, Tabriz, Iran, ORCID ID: 0000-0003-4433-9949, Email: K_shadvar@yahoo.com

**2. Nader Nader-Djalal:** Department of Anesthesiology, Buffalo University, NY, USA, ORCID IDr: 0000-0002-5744-7319, Email: nnader@buffalo.edu

**3. Noushin Vahed:** Faculty of Medicine, Tabriz University of Medical Sciences, Tabriz, Iran, ORCID ID: 0000-0000-0000-0000, Email: vahednooshin@yahoo.com

**4. Sarvin Sanaie:** Neurosciences research center, Aging Research Institute, Tabriz University of Medical Sciences, Iran, ORCID ID: 0000-0003-2325-5631, Email: sarvin_so2000@yahoo.com

**5. Afshin Iranpour:** Al-Zahra private Hospital, Dubai, UAE. ORCID ID: 0000-0002-0013-3992, Email: af6872@yahoo.com

**6. Ata Mahmoodpoor:** Evidence Based Research center, Tabriz University of Medical Sciences, Tabriz, Iran, ORCID ID: 0000-0002-4361-6230, Email: amahmoodpoor@yahoo.com

**7. Abbas Samim:** Chemical Injuries Research Center, Systems Biology and Poisonings Institute, Baqiyatallah University of Medical Sciences, Tehran, Iran. ORCID ID: 0000-0000-0000-0000. E-Mail: int.samim1358@gmail.com

**8. Amir Vahedian-Azimi:** Trauma research center, Nursing Faculty, Baqiyatallah University of Medical Sciences, Tehran, Iran, ORCID ID: 0000-0002-1678- 7608. Email: Amirvahedian63@gmail.com

**9. Farshid Rahimi-bashar:** Anesthesia and Critical Care Department, Hamadan University of Medical Sciences, Hamadan, Iran; ORCID ID: 0000-0001-8276-1425. Email: fr_rahimibashar@yahoo.com

*** Corresponding Authors;** Ata Mahmoodpoor & Farshid Rahimi-bashar

**Table 1:** Univariate and multivariate logistic regression analysis to determine the effect of baseline characteristics and biomarkers on ICU length of stay and RRT

| Variables | Univariate | | Multivariate | |
| --- | --- | --- | --- | --- |
|  | **OR (95% CI)** | ***P*-value** | **OR (95% CI)** | ***P*-value** |
| ICU length of stay (≥11 days vs. <11 days) | |  |  |  |
| Age | 1.245 (1.07-1.997) | 0.038* | 1.648 (0.958-1.047) | 0.288 |
| Gender (Female vs. male) | 0.78 (0.411-1.481) | 0.448 | - | **-** |
| Comorbidity (Yes vs. No) | 2.233 (0.736-6.773) | 0.156 | - | - |
| APACHE IV | 1.421 (1.233-1.638) | <0.001* | 1.807 (1.022-3.195) | **0.042*** |
| SOFA | 1.914 (1.443-2.538) | <0.001* | 0.435 (0.136-1.389) | 0.161 |
| Multiple organ failure (Yes vs. No) | 4.166 (2.059-8.431) | <0.001* | 0.524 (0.113-2.432) | 0.409 |
| Blood culture (Yes vs. No) | 1.261 (0.656-2.423) | 0.487 | - | - |
| Duration of MV | 3.544 (2.278-5.511) | <0.001* | 3.707 (1.948-7.056) | **<0.001*** |
| Duration of using vasopressor | 2.398 (1.783-3.226) | <0.001* | 0.801 (0.403-1.594) | 0.528 |
| RRT (Yes vs. No) | 5.28 (2.134-13.066) | <0.001* | 1.743 (0.363-8.368) | 0.487 |
| Lactate/Albumin | 855314.2  (2005.498-364778527.4) | <0.001* | 5833.219  (0-1.55439×10^16^) | 0.553 |
| Lactate baseline | 0.794 (0.359-1.756) | 0.568 | - | - |
| Lactate at 6h | 1.311 (0.574-2.992) | 0.521 | - | - |
| Lactate at 12h | 1.873 (0.931-3.768) | 0.078 | - | - |
| Lactate at 24h | 3.388 (1.686-6.808) | 0.001* | 3.050 (0.537-17.315) | 0.208 |
| Clearance lactate at 6h | 0.962 (0.913-1.014) | 0.149 | - | - |
| Clearance lactate at 12h | 0.961 (0.929-0.994) | 0.020* | 0.906 (0.3-2.731) | 0.861 |
| Clearance lactate at 24h | 0.942 (0.914-0.971) | <0.001* | 0.936 (0.337-2.598) | 0.898 |
| RRT (Yes vs. No) | |  |  |  |
| Age | 0.982 (0.929-1.039) | 0.531 | - | - |
| Gender (Female vs. male) | 0.684 (0.32-1.462) | 0.327 | - | - |
| Comorbidity (Yes vs. No) | 0.743 (0.199-2.771) | 0.658 | - | - |
| APACHE IV | 1.267 (1.097-1.464) | 0.001 | 1.503 (1.029-2.194) | **0.035*** |
| SOFA | 1.432 (1.09-1.882) | 0.010* | 0.609 (0.283-1.313) | 0.206 |
| Multiple organ failure (Yes vs. No) | 2.5 (1.156-5.408) | 0.020* | 1.669 (0.503-5.542) | 0.403 |
| Blood culture (Yes vs. No) | 1.96 (0.877-4.381) | 0.101 | - | - |
| ICU length of stay | 1.286 (1.133-1.459) | <0.001* | 1.015 (0.778-1.324) | 0.913 |
| Duration of MV | 1.418 (1.212-1.66) | <0.001* | 1.296 (0.839-2.003) | 0.243 |
| Duration of using vasopressor | 1.553 (1.265-1.908) | <0.001* | 1.121 (0.723-1.737) | 0.611 |
| Lactate/Albumin | 62617.8  (200.692-19537427.9) | <0.001* | 7044.081  (0.792-62611291.37) | 0.056 |
| Lactate baseline | 3.366 (1.122-10.098) | 0.030* | 2.259 (0.36-14.154) | 0.384 |
| Lactate at 6h | 3.841 (1.302-11.334) | 0.015* | 0.639 (0.089-4.597) | 0.656 |
| Lactate at 12h | 2.849 (1.268-6.403) | 0.011* | 0.385 (0.065-2.282) | 0.293 |
| Lactate at 24h | 3.929 (1.838-8.398) | <0.001* | 6.335 (1.116-35.975) | **0.037*** |
| Clearance lactate at 6h | 0.964 (0.906-1.027) | 0.261 | 0.994 (0.915-1.079) | 0.879 |
| Clearance lactate at 12h | 0.97 (0.935-1.007) | 0.111 | 0.988 (0.916-1.066) | 0.759 |
| Clearance lactate at 24h | 0.953 (0.923-0.985) | 0.004* | 0.987 (0.918-1.062) | 0.726 |

*P<0.05 considered as significant, Abbreviations: Odds ratio (OR), Confidence Interval (CI)

**Table 2:** Univariate and multivariate logistic regression analysis to determine the effect of baseline characteristics and biomarkers on duration of using vasopressor

| Variables | Univariate | | Multivariate | |
| --- | --- | --- | --- | --- |
|  | **OR (95% CI)** | ***P*-value** | **OR (95% CI)** | ***P*-value** |
| Duration of using vasopressor (≥4 days vs. <4days) | |  |  |  |
| Age | 0.944 (0.887-1.006) | 0.074 | - | - |
| Gender (Female vs. male) | 0.705 (0.347-1.435) | 0.336 | - | - |
| Comorbidity (Yes vs. No) | 3.161 (0.687-14.537) | 0.139 | - | - |
| APACHE IV | 1.394 (1.202-1.618) | <0.001* | 1.084 (0.656-1.792) | 0.752 |
| SOFA | 2.162 (1.539-3.037) | <0.001* | 1.512 (0.513-4.457) | 0.453 |
| Multiple organ failure (Yes vs. No) | 15.898 (4.634-54.547) | <0.001* | 2.098 (1.019-4.546) | **0.005*** |
| Blood culture (Yes vs. No) | 1.186 (0.58-2.423) | 0.641 | - | - |
| ICU length of stay | 2.048 (1.553-2.7) | <0.001* | 0.954 (0.615-1.482) | 0.836 |
| Duration of MV | 3.424 (2.208-5.308) | <0.001* | 3.101 (1.516-6.344) | **0.002*** |
| RRT (Yes vs. No) | 5.831 (1.682-20.214) | 0.005* | 0.502 (0.054-4.692) | 0.546 |
| Lactate/Albumin | 3.9037×10^18^  (20430087223-7.4591×10^24^) | <0.001* | 4.84901×10^13^  (46140.3-5.095×10^22^) | **0.003*** |
| Lactate baseline | 1.043 (0.441-2.469) | 0.923 | 0.228 (0.028-1.884) | 0.171 |
| Lactate at 6h | 2.842 (1.09-7.409) | 0.033* | 0.287 (0.019-4.418) | 0.371 |
| Lactate at 12h | 10.661 (3.596-31.609) | <0.001* | 6.657 (0.265-16.416) | 0.249 |
| Lactate at 24h | 25.566 (6.87-95.133) | <0.001* | 4.846 (0.306-7.768) | 0.263 |
| Clearance lactate at 6h | 0.914 (0.861-0.97) | 0.003* | 1.049 (0.946-1.164) | 0.361 |
| Clearance lactate at 12h | 0.867 (0.817-0.921) | <0.001* | 0.922 (0.817-1.04) | 0.184 |
| Clearance lactate at 24h | 0.882 (0.84-0.925) | <0.001* | 0.948 (0.851-1.056) | 0.334 |

*P<0.05 considered as significant, Abbreviations: Odds ratio (OR), Confidence Interval (CI)

| A   | B   | C   |
| --- | --- | --- |
| D   | E   | F   |
| G   | H   |  |

**Figure 1.** ROC curve of biomarkers for mortality prediction

| A   | B   | C   |
| --- | --- | --- |
| D   | E   | F   |
| G   | H   |  |

**Figure 2.** ROC curve of biomarkers for RRT prediction

| A   | B   | C   |
| --- | --- | --- |
| D   | E   | F   |
| G   | H   |  |

**Figure 3.** ROC curve of biomarkers for ICU LOS prediction

| A   | B   | C   |
| --- | --- | --- |
| D   | E   | F   |
| G   | H   |  |

**Figure 4.** ROC curve of biomarkers for MV duration prediction

| A   | B   | C   |
| --- | --- | --- |
| D   | E   | F   |
| G   | H   |  |

**Figure 5.** ROC curve of biomarkers for duration of using vasopressor prediction

|  | \| Lactate/Albumin vs. Lactate \| \| \| --- \| --- \| \| Difference between areas \| 0.244 \| \| Significance level \| **P < 0.0001*** \| \| Lactate/Albumin vs. Clearance lactate \| \| \| Difference between areas \| 0.214 \| \| Significance level \| **P < 0.0001*** \| \| Lactate/Albumin vs. Combined marker \| \| \| Difference between areas \| 0.0167 \| \| Significance level \| P = 0.1374 \| \| Lactate vs. Clearance lactate \| \| \| Difference between areas \| 0.0304 \| \| Significance level \| P = 0.5793 \| \| Lactate vs. Combined marker \| \| \| Difference between areas \| 0.261 \| \| Significance level \| **P < 0.0001*** \| \| Clearance lactate vs. Combined marker \| \| \| Difference between areas \| 0.230 \| \| Significance level \| **P < 0.0001*** \| |
| --- | --- | --- | --- | --- | --- | --- | --- | --- | --- | --- | --- | --- | --- | --- | --- | --- | --- | --- | --- | --- | --- | --- | --- | --- | --- | --- | --- | --- | --- | --- | --- | --- | --- | --- | --- | --- | --- |

**Figure 6.** Pairwise comparison of ROC curves between markers to predict mortality at 6h

|  | \| Lactate vs. Clearance lactate \| \| \| --- \| --- \| \| Difference between areas \| 0.0418 \| \| Standard Error ^a^ \| 0.0424 \| \| 95% Confidence Interval \| -0.0413 to 0.125 \| \| Significance level \| P = 0.3240 \| \| Lactate vs. Combined marker at 12h \| \| \| Difference between areas \| 0.0422 \| \| Standard Error ^a^ \| 0.0358 \| \| 95% Confidence Interval \| -0.0279 to 0.112 \| \| Significance level \| P = 0.2381 \| \| Clearance lactate vs. Combined marker at 12h \| \| \| Difference between areas \| 0.000434 \| \| Standard Error ^a^ \| 0.00915 \| \| 95% Confidence Interval \| -0.0175 to 0.0184 \| \| Significance level \| P = 0.9622 \| |
| --- | --- | --- | --- | --- | --- | --- | --- | --- | --- | --- | --- | --- | --- | --- | --- | --- | --- | --- | --- | --- | --- | --- | --- | --- | --- | --- | --- | --- | --- | --- | --- |

**Figure 7.** Pairwise comparison of ROC curves between markers to predict mortality at 12h

|  | \| Lactate 24h vs. Clearance lactate 24h \| \| \| --- \| --- \| \| Difference between areas \| 0.0409 \| \| Standard Error ^a^ \| 0.0244 \| \| 95% Confidence Interval \| -0.00686 to 0.0887 \| \| Significance level \| P = 0.0932 \| \| Lactate 24h vs. Combined marker 24h \| \| \| Difference between areas \| 0.0399 \| \| Standard Error ^a^ \| 0.0227 \| \| 95% Confidence Interval \| -0.00445 to 0.0843 \| \| Significance level \| P = 0.0778 \| \| Clearance lactate 24h vs. Combined marker 24 \| \| \| Difference between areas \| 0.000977 \| \| Standard Error ^a^ \| 0.00247 \| \| 95% Confidence Interval \| -0.00387 to 0.00583 \| \| Significance level \| P = 0.69 \| |
| --- | --- | --- | --- | --- | --- | --- | --- | --- | --- | --- | --- | --- | --- | --- | --- | --- | --- | --- | --- | --- | --- | --- | --- | --- | --- | --- | --- | --- | --- | --- | --- |

**Figure 8.** Pairwise comparison of ROC curves between markers to predict mortality at 24h

|  | \| Lactate/Albumin vs. Lactate 6h \| \| \| --- \| --- \| \| Difference between areas \| 0.0718 \| \| Significance level \| P = 0.2456 \| \| Lactate/Albumin vs. Clearance lactate 6h \| \| \| Difference between areas \| 0.118 \| \| Significance level \| P = 0.0500 \| \| Lactate/Albumin vs. Combined marker 6h \| \| \| Difference between areas \| 0.0325 \| \| Significance level \| P = 0.1544 \| \| Lactate 6h vs. Clearance lactate 6h \| \| \| Difference between areas \| 0.0458 \| \| Significance level \| P = 0.4640 \| \| Lactate 6h vs. Combined marker 6h \| \| \| Difference between areas \| 0.104 \| \| Significance level \| P = 0.0627 \| \| Clearance lactate 6h vs. Combined marker 6h \| \| \| Difference between areas \| 0.150 \| \| Significance level \| **P = 0.0327*** \| |
| --- | --- | --- | --- | --- | --- | --- | --- | --- | --- | --- | --- | --- | --- | --- | --- | --- | --- | --- | --- | --- | --- | --- | --- | --- | --- | --- | --- | --- | --- | --- | --- | --- | --- | --- | --- | --- | --- |

**Figure 9.** Pairwise comparison of ROC curves between markers to predict RRT at 6h

|  | \| Lactate 12h vs. Clearance lactate 12h \| \| \| --- \| --- \| \| Difference between areas \| 0.0348 \| \| Standard Error ^a^ \| 0.0452 \| \| 95% Confidence Interval \| -0.0538 to 0.124 \| \| Significance level \| P = 0.4411 \| \| Lactate 12h vs. Combined marker 12h \| \| \| Difference between areas \| 0.0393 \| \| Standard Error ^a^ \| 0.0190 \| \| 95% Confidence Interval \| 0.00220 to 0.0765 \| \| Significance level \| P = 0.0379* \| \| Clearance lactate 12h vs. Combined marker 12h \| \| \| Difference between areas \| 0.0742 \| \| Standard Error ^a^ \| 0.0600 \| \| 95% Confidence Interval \| -0.0434 to 0.192 \| \| Significance level \| P = 0.2164 \| |
| --- | --- | --- | --- | --- | --- | --- | --- | --- | --- | --- | --- | --- | --- | --- | --- | --- | --- | --- | --- | --- | --- | --- | --- | --- | --- | --- | --- | --- | --- | --- | --- |

**Figure 10.** Pairwise comparison of ROC curves between markers to predict RRT at 12h

|  | \| Lactate 24h vs. Clearance lactate 24h \| \| \| --- \| --- \| \| Difference between areas \| 0.0289 \| \| Standard Error ^a^ \| 0.0294 \| \| 95% Confidence Interval \| -0.0288 to 0.0866 \| \| Significance level \| P = 0.3267 \| \| Lactate 24h vs. Combined marker 24h \| \| \| Difference between areas \| 0.00530 \| \| Standard Error ^a^ \| 0.0112 \| \| 95% Confidence Interval \| -0.0167 to 0.0273 \| \| Significance level \| P = 0.6373 \| \| Clearance lactate 24h vs. Combined marker 24h \| \| \| Difference between areas \| 0.0236 \| \| Standard Error ^a^ \| 0.0395 \| \| 95% Confidence Interval \| -0.0537 to 0.101 \| \| Significance level \| P = 0.5500 \| |
| --- | --- | --- | --- | --- | --- | --- | --- | --- | --- | --- | --- | --- | --- | --- | --- | --- | --- | --- | --- | --- | --- | --- | --- | --- | --- | --- | --- | --- | --- | --- | --- |

**Figure 11.** Pairwise comparison of ROC curves between markers to predict RRT at 24h

|  | \| Lactate/Albumin vs. Lactate 6h \| \| \| --- \| --- \| \| Difference between areas \| 0.121 \| \| Significance level \| P = 0.0119* \| \| Lactate/Albumin vs. Clearance lactate 6h \| \| \| Difference between areas \| 0.119 \| \| Significance level \| P = 0.0164* \| \| Lactate/Albumin vs. Combined marker 6h \| \| \| Difference between areas \| 0.0115 \| \| Significance level \| P = 0.4793 \| \| Lactate 6h vs. Clearance lactate 6h \| \| \| Difference between areas \| 0.00114 \| \| Significance level \| P = 0.9799 \| \| Lactate 6h vs. Combined marker 6h \| \| \| Difference between areas \| 0.109 \| \| Significance level \| P = 0.0661 \| \| Clearance lactate 6hvs. Combined marker 6h \| \| \| Difference between areas \| 0.108 \| \| Significance level \| P = 0.0414* \| |
| --- | --- | --- | --- | --- | --- | --- | --- | --- | --- | --- | --- | --- | --- | --- | --- | --- | --- | --- | --- | --- | --- | --- | --- | --- | --- | --- | --- | --- | --- | --- | --- | --- | --- | --- | --- | --- | --- |

**Figure 12.** Pairwise comparison of ROC curves between markers to predict ICU LOS at 6h

|  | \| Lactate 12 h vs. Clearance lactate 12h \| \| \| --- \| --- \| \| Difference between areas \| 0.0253 \| \| Standard Error ^a^ \| 0.0311 \| \| 95% Confidence Interval \| -0.0356 to 0.0862 \| \| Significance level \| P = 0.4156 \| \| Lactate 12h vs. Combined marker 12h \| \| \| Difference between areas \| 0.0267 \| \| Standard Error ^a^ \| 0.0327 \| \| 95% Confidence Interval \| -0.0373 to 0.0908 \| \| Significance level \| P = 0.4134 \| \| Clearance lactate 12h vs. Combined marker 12h \| \| \| Difference between areas \| 0.00143 \| \| Standard Error ^a^ \| 0.00290 \| \| 95% Confidence Interval \| -0.00425 to 0.00710 \| \| Significance level \| P = 0.6222 \| |
| --- | --- | --- | --- | --- | --- | --- | --- | --- | --- | --- | --- | --- | --- | --- | --- | --- | --- | --- | --- | --- | --- | --- | --- | --- | --- | --- | --- | --- | --- | --- | --- |

**Figure 13.** Pairwise comparison of ROC curves between markers to predict ICU LOS at 12h

|  | \| Lactate 24 h vs. Clearance lactate 24h \| \| \| --- \| --- \| \| Difference between areas \| 0.00961 \| \| Standard Error ^a^ \| 0.0207 \| \| 95% Confidence Interval \| -0.0310 to 0.0503 \| \| Significance level \| P = 0.6431 \| \| Lactate 24h vs. Combined marker 24h \| \| \| Difference between areas \| 0.00970 \| \| Standard Error ^a^ \| 0.0209 \| \| 95% Confidence Interval \| -0.0312 to 0.0506 \| \| Significance level \| P = 0.6421 \| \| Clearance lactate 24h vs. Combine marker 24h \| \| \| Difference between areas \| 0.0000951 \| \| Standard Error ^a^ \| 0.000677 \| \| 95% Confidence Interval \| -0.00123 to 0.00142 \| \| Significance level \| P = 0.8883 \| |
| --- | --- | --- | --- | --- | --- | --- | --- | --- | --- | --- | --- | --- | --- | --- | --- | --- | --- | --- | --- | --- | --- | --- | --- | --- | --- | --- | --- | --- | --- | --- | --- |

**Figure 14.** Pairwise comparison of ROC curves between markers to predict ICU LOS at 24h

|  | \| Lactate/Albumin vs. Lactate 6h \| \| \| --- \| --- \| \| Difference between areas \| 0.132 \| \| Significance level \| P = 0.0058 \| \| Lactate/Albumin vs. Clearance lactate 6h \| \| \| Difference between areas \| 0.140 \| \| Significance level \| P = 0.0117 \| \| Lactate/Albumin vs. Combined marker 6h \| \| \| Difference between areas \| 0.00270 \| \| Significance level \| P = 0.8883 \| \| Lactate 6h vs. Clearance lactate 6h \| \| \| Difference between areas \| 0.00776 \| \| Significance level \| P = 0.8650 \| \| Lactate 6h vs. Combined marker 6h \| \| \| Difference between areas \| 0.135 \| \| Significance level \| P = 0.0013 \| \| Clearance lactate 6h vs. Combined marker 6h \| \| \| Difference between areas \| 0.143 \| \| Significance level \| P = 0.0005 \| |
| --- | --- | --- | --- | --- | --- | --- | --- | --- | --- | --- | --- | --- | --- | --- | --- | --- | --- | --- | --- | --- | --- | --- | --- | --- | --- | --- | --- | --- | --- | --- | --- | --- | --- | --- | --- | --- | --- |

**Figure 15.** Pairwise comparison of ROC curves between markers to predict duration of MV at 6h

|  | \| Lactate 12h vs. Clearance lactate 12h \| \| \| --- \| --- \| \| Difference between areas \| 0.0125 \| \| Standard Error ^a^ \| 0.0296 \| \| 95% Confidence Interval \| -0.0454 to 0.0704 \| \| Significance level \| P = 0.6727 \| \| Lactate 12 h vs. Combined marker 12h \| \| \| Difference between areas \| 0.00979 \| \| Standard Error ^a^ \| 0.0211 \| \| 95% Confidence Interval \| -0.0315 to 0.0511 \| \| Significance level \| P = 0.6420 \| \| Clearance lactate 12h vs. Combined marker 12h \| \| \| Difference between areas \| 0.00270 \| \| Standard Error ^a^ \| 0.0113 \| \| 95% Confidence Interval \| -0.0195 to 0.0249 \| \| Significance level \| P = 0.8117 \| |
| --- | --- | --- | --- | --- | --- | --- | --- | --- | --- | --- | --- | --- | --- | --- | --- | --- | --- | --- | --- | --- | --- | --- | --- | --- | --- | --- | --- | --- | --- | --- | --- |

**Figure 16.** Pairwise comparison of ROC curves between markers to predict duration of MV at 12h

|  | \| Lactate 24h vs. Clearance lactate 24h \| \| \| --- \| --- \| \| Difference between areas \| 0.00450 \| \| Standard Error ^a^ \| 0.0181 \| \| 95% Confidence Interval \| -0.0310 to 0.0400 \| \| Significance level \| P = 0.8037 \| \| Lactate 24h vs. Combined marker 24h \| \| \| Difference between areas \| 0.00315 \| \| Standard Error ^a^ \| 0.0118 \| \| 95% Confidence Interval \| -0.0199 to 0.0262 \| \| Significance level \| P = 0.7887 \| \| Clearance lactate 24h vs. Combined marker 24h \| \| \| Difference between areas \| 0.00135 \| \| Standard Error ^a^ \| 0.00844 \| \| 95% Confidence Interval \| -0.0152 to 0.0179 \| \| Significance level \| P = 0.8730 \| |
| --- | --- | --- | --- | --- | --- | --- | --- | --- | --- | --- | --- | --- | --- | --- | --- | --- | --- | --- | --- | --- | --- | --- | --- | --- | --- | --- | --- | --- | --- | --- | --- |

**Figure 17.** Pairwise comparison of ROC curves between markers to predict duration of MV at 24h

|  | \| Lactate/Albumin vs. Lactate 6h \| \| \| --- \| --- \| \| Difference between areas \| 0.112 \| \| Significance level \| P = 0.0226 \| \| Lactate/Albumin vs. Clearance lactate 6h \| \| \| Difference between areas \| 0.102 \| \| Significance level \| P = 0.0460 \| \| Lactate/Albumin vs. Combined marker 6h \| \| \| Difference between areas \| 0.0269 \| \| Significance level \| P = 0.1353 \| \| Lactate 6h vs. Clearance lactate 6h \| \| \| Difference between areas \| 0.0105 \| \| Significance level \| P = 0.8064 \| \| Lactate 6h vs. Combined marker 6h \| \| \| Difference between areas \| 0.139 \| \| Significance level \| P = 0.0011 \| \| Clearance lactate 6h vs. Combined marker 6h \| \| \| Difference between areas \| 0.128 \| \| Significance level \| P = 0.0006 \| |
| --- | --- | --- | --- | --- | --- | --- | --- | --- | --- | --- | --- | --- | --- | --- | --- | --- | --- | --- | --- | --- | --- | --- | --- | --- | --- | --- | --- | --- | --- | --- | --- | --- | --- | --- | --- | --- | --- |

**Figure 18**. Pairwise comparison of ROC curves between markers to predict duration of of using vasopressor at 6h

|  | \| Lactate 12h vs. Clearance lactate 12h \| \| \| --- \| --- \| \| Difference between areas \| 0.0376 \| \| Standard Error ^a^ \| 0.0297 \| \| 95% Confidence Interval \| -0.0207 to 0.0958 \| \| Significance level \| P = 0.2061 \| \| Lactate 12h vs. Combined marker 12h \| \| \| Difference between areas \| 0.0361 \| \| Standard Error ^a^ \| 0.0232 \| \| 95% Confidence Interval \| -0.00939 to 0.0816 \| \| Significance level \| P = 0.1198 \| \| Clearance lactate 12h vs. Combined marker \| \| \| Difference between areas \| 0.00146 \| \| Standard Error ^a^ \| 0.00865 \| \| 95% Confidence Interval \| -0.0155 to 0.0184 \| \| Significance level \| P = 0.8657 \| |
| --- | --- | --- | --- | --- | --- | --- | --- | --- | --- | --- | --- | --- | --- | --- | --- | --- | --- | --- | --- | --- | --- | --- | --- | --- | --- | --- | --- | --- | --- | --- | --- |

Figure 19. Pairwise comparison of ROC curves between markers to predict duration of of using vasopressor at 12h

|  | \| Lactate 24h vs. Clearance lactate 24h \| \| \| --- \| --- \| \| Difference between areas \| 0.00844 \| \| Standard Error ^a^ \| 0.0186 \| \| 95% Confidence Interval \| -0.0281 to 0.0450 \| \| Significance level \| P = 0.6508 \| \| Lactate 24h vs. Combined marker 24h \| \| \| Difference between areas \| 0.00709 \| \| Standard Error ^a^ \| 0.0115 \| \| 95% Confidence Interval \| -0.0154 to 0.0296 \| \| Significance level \| P = 0.5373 \| \| Clearance lactate 24h vs. Combined marker 24 \| \| \| Difference between areas \| 0.00135 \| \| Standard Error ^a^ \| 0.00901 \| \| 95% Confidence Interval \| -0.0163 to 0.0190 \| \| Significance level \| P = 0.8809 \| |
| --- | --- | --- | --- | --- | --- | --- | --- | --- | --- | --- | --- | --- | --- | --- | --- | --- | --- | --- | --- | --- | --- | --- | --- | --- | --- | --- | --- | --- | --- | --- | --- |

**Figure 20.** Pairwise comparison of ROC curves between markers to predict duration of of using vasopressor at 24h
